# *RECQL4* alterations in gliomas and nerve sheath tumors: Expression patterns and therapeutic implications

**DOI:** 10.1093/jnen/nlaf129

**Published:** 2025-11-29

**Authors:** Sarra Belakhoua, Gianluca Lopez, Swati Dubey, Simran Rai, Liam Chen, Suping Chen, Aparna Pallavajjala, Ming Yuan, Melike Pekmezci, Marija Stojanova, Christopher M Heaphy, Charles G Eberhart, Fausto J Rodriguez

**Affiliations:** Department of Pathology, New York University, New York City, NY, United States; Department of Pathology and Laboratory Medicine, University of California, Los Angeles, CA, United States; Division of Pathology, Fondazione IRCCS Ca’ Granda—Ospedale Maggiore Policlinico, Milan, Italy; Department of Biomedical, Surgical and Dental Sciences, University of Milan, Milan, Italy; Department of Pathology and Laboratory Medicine, University of California, Los Angeles, CA, United States; Department of Pathology and Laboratory Medicine, University of California, Los Angeles, CA, United States; Department of Pathology, University of Minnesota, Minneapolis, MN, United States; Department of Pathology, Johns Hopkins University, Baltimore, MD, United States; Department of Pathology, Johns Hopkins University, Baltimore, MD, United States; Department of Pathology, Johns Hopkins University, Baltimore, MD, United States; Department of Pathology, University of California, San Francisco, San Francisco, CA, United States; Department of Medicine, Boston Medical Center, Boston, MA, United States; Boston University Chobanian & Avedisian School of Medicine, Boston, MA, United States; Department of Medicine, Boston Medical Center, Boston, MA, United States; Boston University Chobanian & Avedisian School of Medicine, Boston, MA, United States; Department of Pathology, Johns Hopkins University, Baltimore, MD, United States; Department of Pathology, New York University, New York City, NY, United States

**Keywords:** alternative lengthening of telomeres, ATR inhibitors, gliomas, neurofibromatosis type 1, RecQ helicases

## Abstract

RECQL4 plays an important role in maintaining the integrity of the genome and regulating DNA replication. However, the role of RECQL4 in CNS tumors remains unknown. Sequencing data were reviewed and immunohistochemistry was performed on a variety of glial and nerve sheath tumors. Functional studies were performed in glioma (U251) and malignant peripheral nerve sheath tumors (MPNSTs) (NF90-8, ST88-14) cell lines following *RECQL4* knockdown and treatment with ATR-inhibitors. Across 1580 CNS tumors, *RECQL4* gene variants were identified in 71 cases (4.5%), with 21 (29.6%) of probable pathogenic significance. *RECQL4* expression differed significantly across glioma subgroups (*P* = 0.012). Low-grade gliomas (diffuse: median H-score 57.5; circumscribed: median 130) showed lower expression than high-grade gliomas (median 145, *P* < 0.05). Neurofibromas displayed higher RECQL4 expression (median 160) compared with MPNSTs (median 97.5, *P* < 0.001). Among MPNSTs, NF1-associated cases (*n* = 24, median 95) expressed significantly less RECQL4 than sporadic cases (*n* = 8, median 162.5, *P* < 0.001). *RECQL4* knockdown in glioma and MPNST cell lines resulted in increased apoptosis and susceptibility to ATR-inhibitors. Our findings show that RECQL4 expression has divergent patterns across tumor types and that targeting RECQL4 may dampen tumor survival and enhance susceptibility to ATR inhibitor therapy in CNS tumors.

## INTRODUCTION

RECQL4, a ubiquitously expressed protein that belongs to the RecQ Helicase family (which also includes RECQL1, BLM, WRN, and RECQL5), plays a role in maintaining the integrity of the genome and regulating DNA replication.^1^^,^^2^ The *RECQL4* gene is found on 8q24.3, and its protein product contains an N-terminal helicase domain and a C-terminal Drc1-Sld2 domain that interacts with Cycline D2 to initiate DNA replication, contributing to DNA repair (nonhomologous end joining) and to telomere maintenance.^3^^,^^4^ Germline pathogenic variants in the *RECQL4* gene are associated with Rothmund-Thomson syndrome, Baller-Gerold syndrome, and RAPADILINO syndrome,^1^^,^^2^^,^^5^ which share common features such as skeletal abnormalities, changes in skin pigmentation, premature aging, and notably predisposition to cancer, particularly osteosarcoma. Although cancer is the leading cause of death in these patients, there is no increased risk for tumors of the central or peripheral nervous system.

Given its role in genome maintenance and evidence of increased cancer susceptibility in patients with pathogenic germline variants, RECQL4 possibly plays a role in cancer development, although the extent of its contribution and the proportion of human malignancies that rely on alterations of this gene are still poorly defined topics.^2^ In addition, RECQL4 is considered a potential molecular target for cancer therapy given that it correlates with a stem cell phenotype,^6^^,^^7^ and its overexpression has been observed to confer resistance to chemotherapy regimens in preclinical settings, including glioma.^8–10^

Our prior work reported *RECQL4* gene variants in NF1-associated tumors, at times in association with abnormal telomeres.^11^ However, the relevance of *RECQL4* gene variants in gliomas and neurofibromatosis-associated neoplasms is unclear, and a possible relation to the alternative lengthening of telomeres (ALT) is currently unproven. This work aims to investigate the genetic alterations and protein expression of RECQL4 in gliomas and neurofibromatosis type 1-associated neoplasms and their correlation to ALT and related molecular mechanisms and to assess the role of RECQL4-knockdown cells in survival and susceptibility to ATR-kinase inhibitors.

## METHODS

### cBioPortal analysis

A cBioPortal analysis was performed to assess the frequency of *RECQL4* alterations both in cases of gliomas (including low-grade and high-grade) and peripheral nerve sheath tumors. The glioma analysis included cases from 10 different studies and a total of 4206 samples/3453 patients (https://bit.ly/3V5sCKC). The peripheral nerve sheath tumor analysis included cases from a single study and a total of 80 samples/51 patients (https://bit.ly/3V5BRKX).

### Next-generation sequencing

Human tumor samples were gathered at Johns Hopkins Hospital with local Institutional Review Board authorization and consent or waiver of consent when appropriate. The tissues underwent mincing and digestion using either papain dissociation system (Grand Island, New York, NY, USA) followed by filtration through a 70-μm Falcon cell strainer (ThermoFisher Scientific, Waltham, MA, USA).

Next-generation sequencing (NGS) was performed with the Illumina panel at Johns Hopkins Hospital using methods previously described in the literature and molecular pathology website. In brief, areas enriched with tumor (at least 10% tumor cellularity) were scraped from sequential formalin-fixed paraffin-embedded sections 5-μm-thick (routinely 3 to 7 unstained slides per sample) and placed into 2 ml tubes for DNA extraction. Extraction and purification were accomplished with the automated Siemens Tissue Preparation System (Siemens Healthcare Diagnostics, Inc., Tarrytown, NY, USA); genomic DNA was quantified using the Qubit 2.0 Fluorometer (Life Technologies, Carlsbad, CA, USA). DNA libraries were prepared using Agilent SureSelect-XT reagents (Agilent Technologies, Inc., Santa Clara, CA). A hybrid-capture approach was used to target genomic DNA regions of interest by means of an Agilent custom-designed bait set covering the full coding regions of 644 cancer-associated genes (core list of genes available at the Johns Hopkins Molecular Pathology website: https://pathology.jhu.edu/build/assets/testDirectory/SolidTumorPanel-II_GeneList_v9.pdf). Sequencing of libraries was performed to an average unique read depth of greater than 500 × using Sequencing by Synthesis 2 × 100 base pairs paired-end cluster generation on the Illumina HiSeq 2500 platform (Illumina, Inc., San Diego, CA, USA). FASTQ files were generated from Binary Cluster Files (.bcl) using the Illumina bcl2fastq v1.8.4 software with parameters set as per vendor’s specifications. Alignment to the human genome reference hg19 (GRCh37) was performed using the Burrows-Wheeler Aligner v0.7.10 algorithm with default settings. BAM files (.bam) were generated using Picard Tools v1.119 and variant calling was performed using in-house variant caller algorithm (MDLVC v5.0) cross referenced with HaplotypeCaller (Genome Analysis Tool Kit 3.3) under discovery mode in the coding regions of target genes. All variant calls detected with the bioinformatic approach were manually reviewed and analyzed using Integrated Genomics Viewer v2.3.4 (IGV; Broad Institute, MIT Harvard, Cambridge, MA, USA) and annotated with dbSNP v150 and COSMIC v82 databases. For clinical work, VAF >5% are generally reportable. Common single-nucleotide polymorphisms (population allele frequency > 1%) were excluded from analysis. Matched normal (tissue or peripheral blood) was not available for comparison. The online database ClinVar was queried for all alterations to gather information about their biological and clinical significance. Gene variants that were likely SNPs based on public databases, and those with variant allele frequency <5%, between 45% and 55%, and >95% without convincing pathogenicity were excluded.

### ALT validation

ALT was evaluated using methods established in prior publications.^12^ Ultra-bright telomeric foci were examined through telomere-specific FISH. Telomeric extrachromosomal circles, such as C-circles, were identified through immunoblotting following amplification of C-circle DNA using processive phi29 polymerase. A DIG-conjugated probe containing the C-rich telomere repeat sequence was utilized to specifically detect the polymerase amplified signal. The ALT-positive osteosarcoma cell line U2OS, whose ALT status is known, was employed as a positive control.

### Immunohistochemistry evaluation of RECQL4 expression

Immunohistochemistry (IHC) with anti-RECQL4 antibody (Proteintech 17008-1-AP, 1:50) was utilized on a tissue microarray (TMA) consisting of 21 low-grade gliomas ([LGG]; 17 low-grade circumscribed gliomas [LGCG] and 4 low-grade diffuse gliomas [LGDG] all of which were angiocentric gliomas, AG); 41 adult-type diffuse high-grade gliomas ([HGG], grade 4); and 99 nerve sheath tumors, comprising 67 neurofibromas and 32 malignant peripheral nerve sheath tumors (MPNST). HGGs were stratified for IDH and ATRX status assessed with IHC, and ALT status as detailed below. RECQL4 immunoreactivity was assessed by one of us (F.J.R.) using H-scores (H) (0–200), which were obtained by multiplying intensity of stain (0: no stain, 1: weak stain, and 2: strong stain) by percentage (0–100) of neoplastic cells showing the staining intensity.

### Tissues and cell lines

U251, initially derived from an adult glioblastoma patient, was utilized. U251 cells were cultured in DMEM/F12 supplemented with 10% FBS. MPNST-derived cell lines NF90-8 and ST88-14 were maintained in RPMI supplemented with 10% FBS. All cells were grown in a 37°C humidified incubator with 5% CO_2_. Regular mycoplasma testing was performed, and human cell line identities were confirmed by short tandem repeat (STR) profiling.

### Gene knockdown of RECQL4

Guide RNAs (gRNAs) directed towards *RECQL4* were sourced from Abmgood. Short hairpin RNA sequences targeting human *RECQL4* (sh2 and sh5), along with the vector control pLKO.1, were acquired from Millipore Sigma. Lentiviruses were generated by transfecting 293 T cells with shRNA or gRNA plasmids along with VSVG packaging plasmids using lipofectamine 2000. After 48–72 h, lentiviral supernatants were harvested and stored at -80°C until required. Cells infected with the virus were subjected to selection with puromycin (1–2 μg/mL) for 7 days to establish stable cell lines.

### Western blotting

Western blotting was utilized for RECQL4 knockdown validation. Cells were lysed using RIPA lysis buffer supplemented with protease inhibitors from MilliporeSigma. The primary antibody utilized for Western blot analysis was RECQL4 (17008-1-AP, 1:2000, Proteintech, Rosemont, IL, USA), the secondary antibodies used was anti-rabbit IgG HRP-linked (#7074, 1:5000, Cell Signaling Technology).

### Cell growth assessment

To evaluate the impact on cell growth, the CellTiter-Blue assay from Promega was employed. Briefly, 1000 to 5000 cells were seeded in triplicate in 96-well plates. Subsequently, at chosen time points, 20–30 μL of the CellTiter-Blue reagent was added to each well and incubated for 1–4 h at 37°C in 5% CO_2_. For drug treatments, cells in 96-well plates were exposed to various concentrations of AZD6738, VE-821, or temozolomide obtained from Selleck Chemicals for 7 days. Vehicle-treated cells (using Dimethyl sulfoxide) served as controls, and the cell survival fraction was determined as a percentage of control cells. Fluorescence (measured at 560 nm excitation/590 nm emission) was quantified using a TECAN plate reader. Additionally, apoptosis assays were conducted using Muse Annexin V and Dead Cell reagent from MilliporeSigma, while bromodeoxyuridine (BrdU) incorporation assays were performed as previously described. Data were collected using a Muse flow cytometer from Millipore and analyzed using FlowJo software.

### Statistical analysis

For cell culture and functional assessments, results were depicted as mean ± SD, with statistical significance denoted by *P* < 0.05. Each experiment was conducted in a minimum of 3 biological replicates, and data were analyzed using either a 2-tailed Student t-test or ANOVA, depending on appropriateness using standard software and statistical packages (GraphPad, San Diego, CA, USA). For IHC scores, group differences were assessed using nonparametric tests: Kruskal–Wallis with pairwise Wilcoxon rank-sum tests (Holm correction) for ≥3 groups, and Wilcoxon rank-sum tests (2-sided) for 2 groups. Analyses and figures were performed in R version 4.5.0 (2025-04-11) with ggplot2; *P* < 0.05 was considered significant.

## RESULTS

### RECQL4 genetic alterations in cBioPortal

Across 10 different queried studies on cBioPortal, genetic alterations in *RECQL4* were found in 86/3453 (2.5%) patients with glioma (92/4206 samples, 2.2%). An Oncoprint representation of the analysis is shown in Figure S1. Details regarding the studies included and the different subtypes of gliomas analyzed are shown in Figures S2 and S3. The different types of genetic alterations in *RECQL4* encountered in this analysis are shown in Figure S4. Among 59 annotated coding mutations, 5 were labeled as pathogenic mutations (1 truncating and 4 splice). All other 54 variants of unknown significance were missense variants. An overall survival analysis comparing cases with *RECQL4* alterations to *RECQL4*-wildtype cases is shown in Figure S5. Interestingly, a better overall survival was noted for cases with *RECQL4* alterations (median overall survival: 25.5 months in RECQL4-wildtype cases, 65.0 months in RECQL4-altered cases; log-rank test *P*-value = 0.006).

Genetic alterations in *RECQL4* were found in 2/51 (3.9%) patients with peripheral nerve sheath tumors (2/80 samples, 2.5%); all of them were missense mutations of unknown significance. All cases with *RECQL4* alterations in this cohort were neurofibromas; there were no *RECQL4* alterations in cases of MPNST.

### RECQL4 gene variants occur in a subset of nervous system tumors

A total of 1580 nervous system tumors underwent NGS and results are summarized in Table 1. Among these, 71 (4.5%) had gene variants in *RECQL4*, of which 21 (29.6%) were of probable pathogenic significance based on the type of variants and/or on data found on public databases (ClinVar, COSMIC). In particular, 3 (14.3%) were frameshift variants, 2 (9.5%) were splice variants (also reported as likely oncogenic on online databases), 2 (9.5%) were duplications, 1 (4.8%) was an insertion, and 13 (61.9%) were point alterations reported to be likely oncogenic on online databases, including one case of a nerve sheath tumor. All other changes observed were missense variants of unknown significance or likely benign.

**Table 1.** RECQL4 alterations.

Diagnosis	*RECQL4* variant	VAF	Reference	Other coding mutations	CNV
Nerve sheath tumor	p.V574I	54.05	COSM9885596		
Astrocytoma	p.A746T	13.20	COSM5457387	*IDH1*, *ATRX*, *PTCH1*, *ATM*	
Astrocytoma	p.N616S	48.82	COSM9351440	*FGFR1*, *PIK3R1*	
Astrocytoma	p.R1140H	48.16	COSM1240034	*EGFR*, *MET*, *NF1*, *TSC1*	Chr 12 amp
Astrocytoma	p.R784Q	51.50	COSM7449791	*IDH1*, *TP53*, *BLM*	
Glioblastoma	c.2464-1G>C	48.85	COSM6986802	*EGFR*, *PTEN*	*EGFR* amp
Glioblastoma	p.R1140H	48.02	COSM1240034	*EGFR*, *MET*, *NF1*, *TSC2*, *CCND2*	*CDK4* amp, *PALB2* amp
Glioma NOS	c.2755 + 1G>A	52.76	ClinVar: 653676	*pTERT*, *TP53*, *TSC2*, *PIK3CA*, *KIT*	*PDGRA* amp
Glioma NOS	p.D1142H	47.62	COSM2871860	*EGFR*, *MSH6*, *PTEN*	Chromosome 4 amp
Glioma NOS	p.M989I	47.10	COSM9496698	*NF2*	
Glioma NOS	p.M989I	52.11	COSM9496698	*IDH1*, *CIC*, *BLM*	
Glioma NOS	p.R1058G	51.18	COSM6910230		
Meningioma G2	p.N616S	13.03	COSM9351440	*CTNBB1*, *BLM*	
Neuroendocrine Neoplasm	p.R1005W	48.74	COSM6924742	*MEN1*	
Oligodendroglioma	p.N616S	53.76	COSM9351440	*IDH1*, *TERT*, *CIC*	
Astrocytoma	p.H831fs*52	50.62	\	*IDH1*, *TP53*, *ATRX*, *FANCA*	
Ependymoma	p.C857_T858dup	26.93	\	*PDGFRA*	
Glioblastoma	p.C525fs	41.84	\	*pTERT*	*EGFR* amp
Glioblastoma	p.T858_R859insCT	25.83	\	*TP53*, *BRCA1*, *HRAS: G12D fusion*	
Glioma NOS	p.L924fs	30.51	\	*NF1*, *ATM*, *PIK3CA*	
Meningioma G1	p.C857_T858dup	34.38	\	*NF2*	

Among nervous system tumors cases from Johns Hopkins Hospital that underwent NGS sequencing from 2017 to 2021 (*n* = 1580), 71 (4.5%) had alterations in *RECQL4*. Among these, 13 point-variants were reported to be likely oncogenic on online databases, as well as 2 splice alterations. The other 8 cases had other types of alterations presumably also oncogenic due to probable loss-of-function significance (total likely oncogenic 21, 29.6%).

### RECQL4 positivity is highest in adult-type high-grade gliomas but varies in subgroups

**Figure 1.**
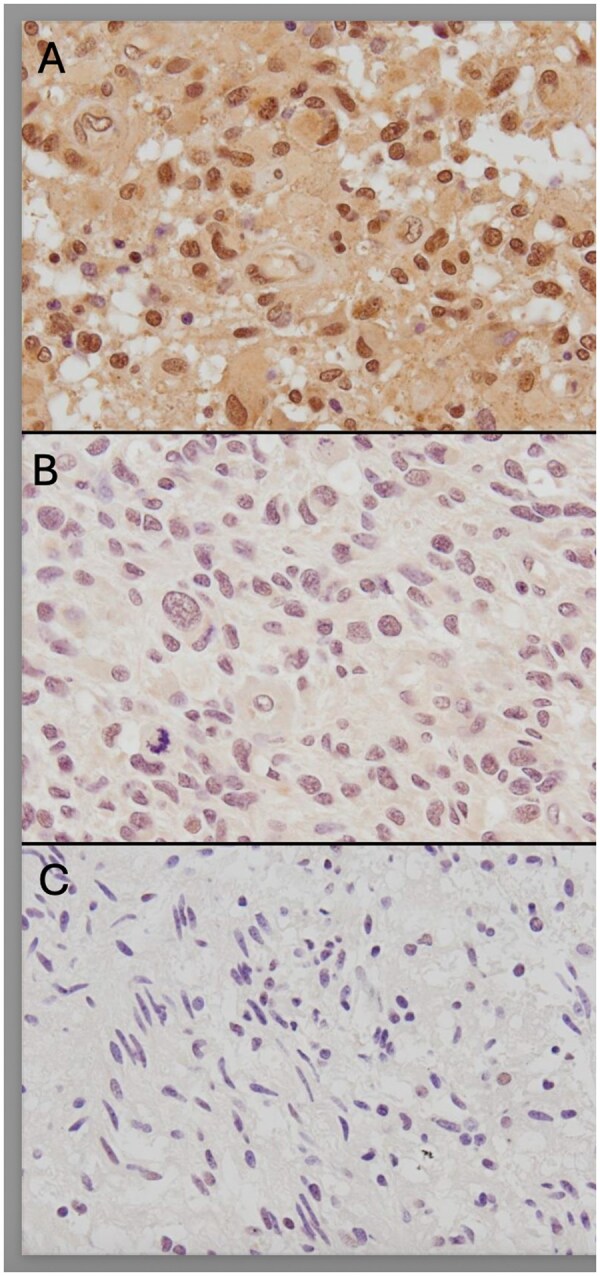
RECQL4 immunohistochemistry. Representative microphotographs of RECQL4 immunohistochemistry staining. (A) HGG with high RECQL4 expression (H-score: 180). (B) HGG with intermediate RECQL4 expression (H-score: 80). (C) Angiocentric glioma with low RECQL4 expression (H-score: 10). HGG, adult-type diffuse high-grade glioma.

**Figure 2.**
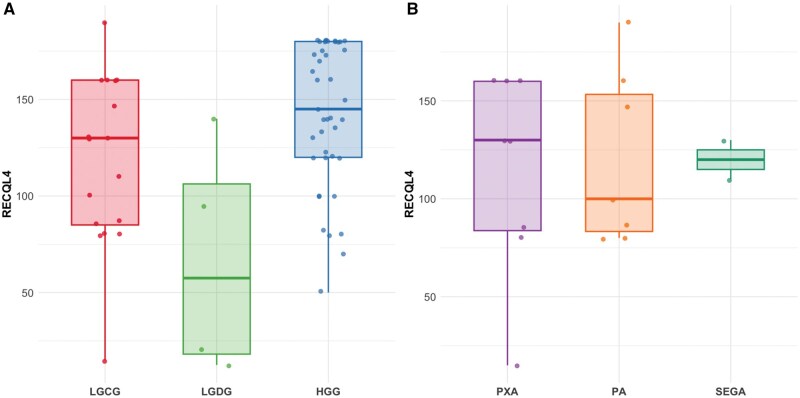
RECQL4 immunohistochemistry in gliomas. (A) RECQL4 immunohistochemical H-scores in low-grade circumscribed glioma (LGCG, *n* = 42), low-grade diffuse glioma (LGDG, *n* = 4), and high-grade glioma (HGG, *n* = 65); expression differed significantly across the groups (Kruskal–Wallis test, χ^2^ = 8.78, df = 2, *P* = 0.012). (B) RECQL4 immunohistochemical H-scores in low-grade circumscribed glioma subtypes, namely subependymal giant-cell astrocytoma (SEGA, *n* = 2), pilocytic astrocytoma (PA, *n* = 7), and pleomorphic xanthoastrocytoma (PXA, *n* = 8). H-scores were similar across groups (Kruskal–Wallis test, χ^2^ = 0.003, df = 2, *P* = 0.999).

Representative microphotographs of RECQL4 IHC are illustrated in Figure 1. Results of RECQL4 H-scoring in gliomas are shown in Tables 2 and 3. Results of RECQL4 IHC scoring in LGG subcategories and HGG are presented in Figure 2A. Overall, LGGs had a median H-score of 110. LGCG had a median H-score of 130. LGDG (AG only, *n* = 4) had a median H-score of 57.5, lower than HGG (*n* = 41) which had in comparison a median of 145. Expression differed significantly across the 3 groups (χ^2^ = 8.78, df = 2, *P* = 0.012). Statistical significance was also observed by comparing LGG as a whole to HGG (W = 251, *P* = 0.0075). H-scores of subcategories of LGCG, including subependymal giant cell astrocytoma ([SEGA], *n* = 2), pilocytic astrocytoma (PA, *n* = 7), and pleomorphic xanthoastrocytoma ([PXA], *n* = 8) are shown in Figure 2B. H-scores were similar across groups (Kruskal–Wallis test, χ^2^ = 0.003, df = 2, *P* = 0.999). TMA RECQL4 H-scores for subcategories of HGGs (stratified for IDH, ALT, and ATRX status) are represented in Table 3. Median H-scores ranged from 140 to 180, without statistically significant differences among different subgroups compared to one another (Figure S6).

**Table 2.** TMA RECQL4 H-scores in 21 low-grade gliomas.

	*N*	Median	Mean ± SD
LGCG (PA+PXA+SEGA)	17	130	117.8 ± 43.9
LGDG (AG)	4	57.5	66.9 ± 61.4
PA	7	100	120.5 ± 44.6
PXA	8	130	115.0 ± 51.6
SEGA	2	120	120.0 ± 14.1
Total	21	110	108.1 ± 50.3

Abbreviations: PA, pilocytic astrocytoma; PXA, pleomorphic xanthoastrocytoma; SEGA, subependymal giant-cell astrocytoma; LGCG, low-grade circumscribed gliomas; LGDG, low-grade diffuse gliomas; AG, angiocentric glioma.

**Table 3.** TMA RECQL4 H-scores in 41 high-grade, adult type gliomas.

	*N*	Median	Mean ± SD
IDH1+	7	165	151.2 ± 35.8
IDH1-	34	140	140.9 ± 37.5
ALT+	10	177	159.8 ± 32.4
ALT-	31	140	137.2 ± 37.2
ATRX+	25	140	140.2 ± 39.1
ATRX-	15	165	149.6 ± 33.3
ALT+/ATRX+	1	180	180.0
ALT+/ATRX-	9	173	157.6 ± 33.5
IDH-/ALT+/ATRX+	1	180	180.0
IDH-/ALT+/ATRX-	2	180	180.0 ± 0.0
Total	41	145	142.7 ± 37.0

ALL IDH+ cases were ATRX+.

Abbreviation: ALT, alternate lengthening of telomeres.

### RECQL4 expression is higher in neurofibromas than MPNST among nerve sheath tumors

**Figure 3.**
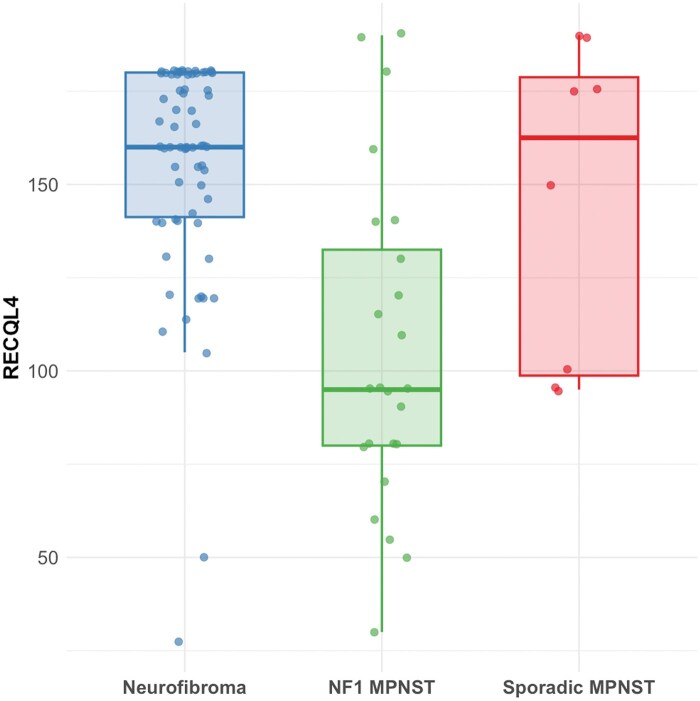
RECQL4 immunohistochemistry in nerve sheath tumors. RECQL4 H-scores across cases of neurofibromas (*n* = 67), NF1-related malignant peripheral nerve sheath tumors (MPNST, *n* = 24), and sporadic MPNST (*n* = 8). Global comparison performed using Kruskal–Wallis test (χ² = 20.54, df = 2, *P* = 0.000035).

RECQL4 scoring in 67 neurofibromas and 32 MPNSTs are presented in Figure 3 and Table 4. RECQL4 expression was significantly higher in neurofibromas (median H-score: 160) compared with MPNST (median H-score: 97.5, Wilcoxon rank-sum test, W = 1590, *P* < 0.0001). A statistical significance was also observed by comparing neurofibromas, NF1-associated MPNST (*n* = 24, median H-score 95) and sporadic MPNST (*n* = 8, median H-score 162.5; χ^2^ = 20.54, df = 2, *P* < 0.0001). Among MPNSTs, ALT-positive cases (*n* = 5) had no significant differences when compared to (median 95) ALT-negative cases (*n* = 25, median 110, *P* > 0.05). Among ALT-positive MPNST cases, RECQL4 H-scores were not statistically different in the setting of ATRX retention (*n* = 3, median 80) when compared with cases with ATRX loss (*n* = 2, median 107.5, *P* > 0.05).

**Table 4.** RECQL4 H-scores in 67 neurofibromas and 32 malignant peripheral nerve sheath tumors (MPNST).

	*N*	Median	**Mean** ± **SD**
Neurofibromas	67	160	157.8 ± 25.8
MPNST	32	97.5	115.6 ± 46.5
NF1 MPNST	24	95	105.4 ± 43.8
Sporadic MPNST	8	162.5	146.3 ± 42.9
ALT+ MPNST	5	95	88.0 ± 25.6
ALT- MPNST	25	110	119.0 ± 47.5
ALT+ ATRX+ MPNST	3	80	75.0 ± 22.9
ALT+ ATRX- MPNST	2	107.5	107.5 ± 17.7

Abbreviation: ALT, alternate lengthening of telomeres.

### RECQL4 loss results in decreased cell growth in glioma and MPNST cell lines

**Figure 4.**
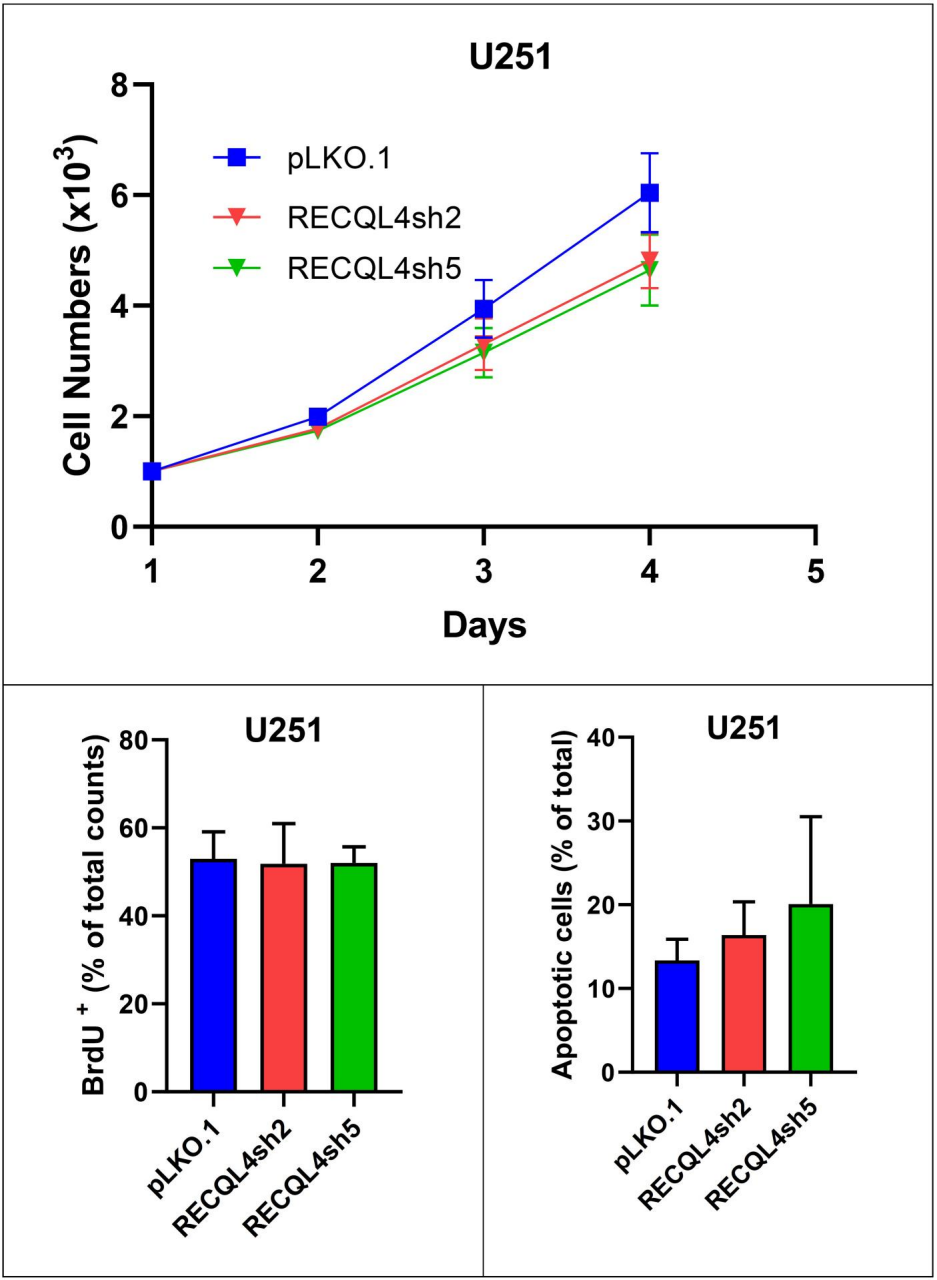
RECQL4 knockdown in glioma. Glioma cell line U251 with its RECQL4-knockdowns (RECQL4sh2 and RECQL4sh5) and empty vector control (pLKO.1). Cell numbers measured at multiple time points (upper). Percentage of BrdU-positive cells (lower left). Percentage of apoptotic cells (lower right).

**Figure 5.**
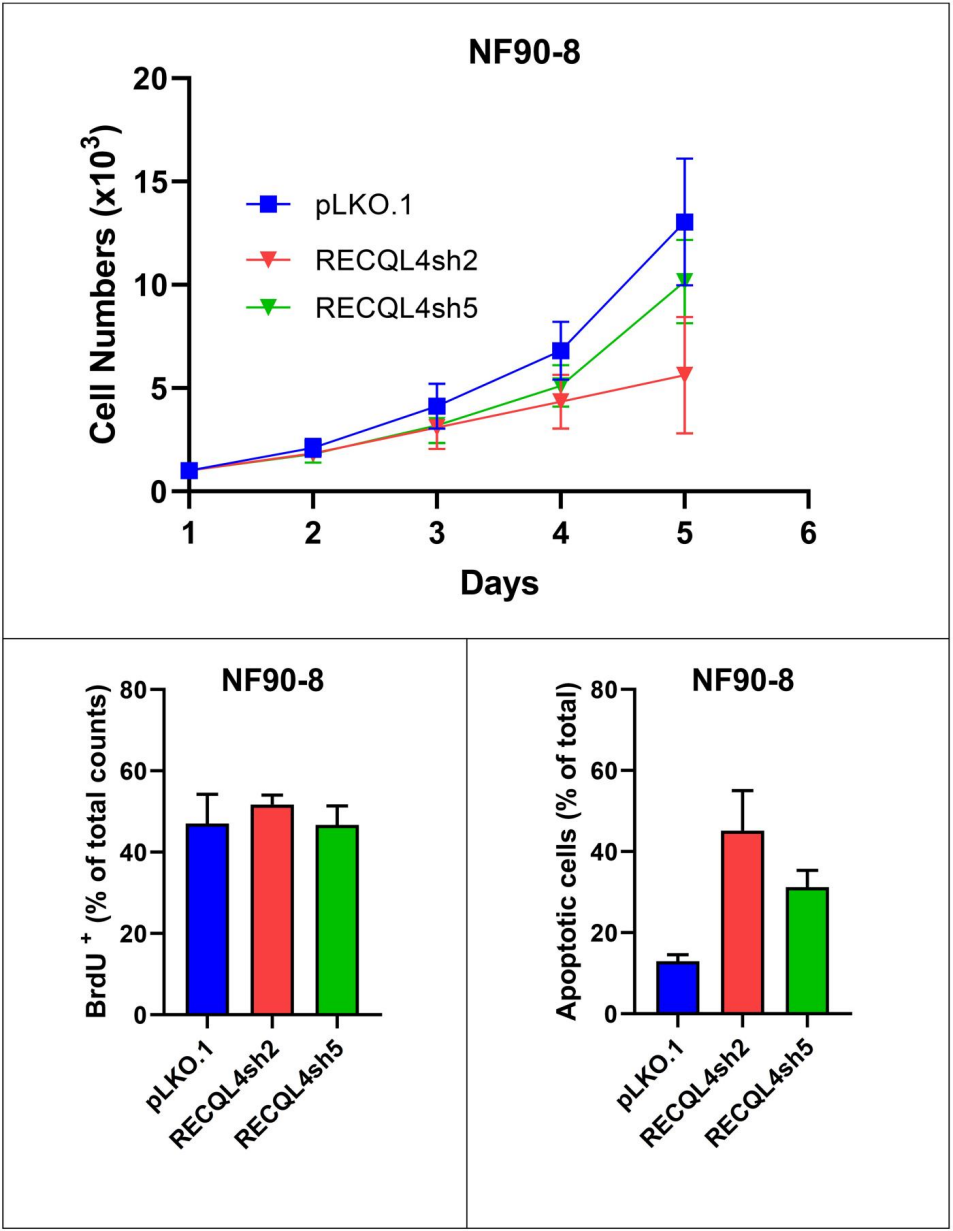
RECQL4 knockdown in MPNST. Malignant peripheral nerve sheath tumor cell line NF90-8 with its RECQL4-knockdowns (RECQL4sh2 and RECQL4sh5) and empty vector control (pLKO.1). Cell numbers measured at multiple time points (upper). Percentage of BrdU-positive cells (lower left). Percentage of apoptotic cells (lower right).

**Figure 6.**
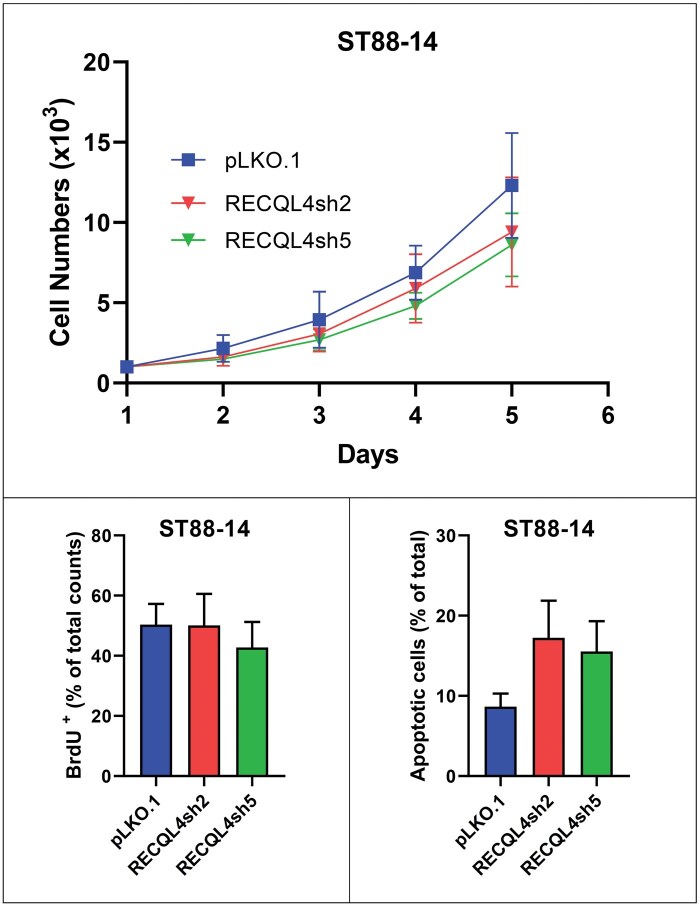
*RECQL4* knockdown in MPNST. Malignant peripheral nerve sheath tumor cell line ST88-14 with its RECQL4-knockouts (RECQL4sh2 and RECQL4sh5) and empty vector control (pLKO.1). Cell numbers measured at multiple time points (upper). Percentage of BrdU-positive cells (lower left). Percentage of apoptotic cells (lower right).

Proliferation and apoptosis data in glioma (U251) and MPNST (NF90-8, ST88-14) cells with *RECQL4* knockdown verified via western blotting (RECQL4sh2 and RECQL4sh5) and controls (pLKO.1) are summarized in Figures 4-6. Knockdown cells exhibited a modest reduction in proliferation rate compared to control cells, as evidenced by lower total cell numbers on day 4 of culture (Figure 4A, 5A, and 6A). Despite this, the proportion of BrdU-positive proliferating cells remained comparable between knockdown and control groups (Figure 4B, 5B, and 6B), indicating that the actively cycling population was not significantly affected. Notably, the percentage of apoptotic cells was significantly higher in the knockdown group, with the most pronounced effect observed in the RECQL4sh5 cell lines (Figure 4C, 5C, and 6C), suggesting that increased cell death might have contributed to the reduced cell numbers observed. In addition, a marked elevation in apoptosis was particularly evident in the NF90-8, RECQL4sh2 cells (Figure 5C).

**Figure 7.**
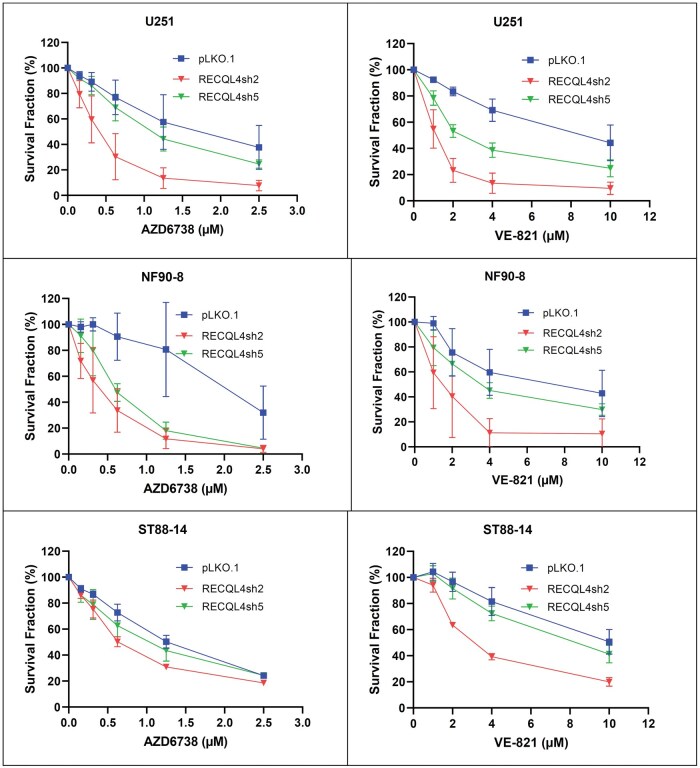
ATR inhibition decreases cell growth in tumor cells with *RECQL4* loss. Effect of ATR kinase inhibition on cell proliferation of glioma and malignant peripheral nerve sheath tumor (MPNST) cell lines and their RECQL4-knockouts. Glioma U251 cell line survival fraction at different dosages of AZD6738 (Upper left) and VE-821 (Upper right). MPNST NF90-8 cell line survival fraction at different dosages of AZD6738 (Mid left)) and VE-821 (Mid right). MPNST ST88-14 cell line survival fraction at different dosages of AZD6738 (Lower left) and VE-821 (Lower right)).

Treatment with ATR kinase inhibitors (AZD6738 and VE-821) led to a marked reduction in the survival fraction across all tested cell lines. However, RECQL4-knockdown cells exhibited greater sensitivity to both inhibitors compared to the corresponding pLKO.1 control cells (although the magnitude of this effect varied among different cell lines) (Figure 7). While increasing concentrations of ATR inhibitors progressively decreased cell survival in all groups, *RECQL4*-knockdown cells demonstrated a more pronounced reduction in survival even at lower drug concentrations.

## DISCUSSION

To investigate the role of RECQL4 in gliomas/neurofibromatosis type 1 associated neoplasms and susceptibility to treatment, we evaluated the prevalence of *RECQL4* genetic alterations in a large cohort of patients with nervous system tumors, assessed the expression of the protein in TMAs, and correlated with clinical, pathological, and molecular aspects, and finally, we assessed the impact of *RECQL4*-knockdown in survival and vulnerability to ATR-kinase inhibitors in related cell lines (Figure S7).

We found *RECQL4* gene variants in 4.5% nervous system tumor cases, and a proportion (29.6%) of these of probable pathogenic (loss of function) significance. Deep deletions were rare and only detected in less than 1% of diffuse gliomas in the TCGA dataset. With the data we have is not possible to know whether complete inactivation or haploinsufficiency is the putative mechanism of tumorigenesis of *RECQL4*. However, data in genetic syndromes and other tumors support a loss of function mechanism.

Increased RECQL4 expression has been reported in many human malignant tumors,^13^^,^^14^ including glioblastoma.^10^ In our analysis, RECQL4 expression showed divergent patterns across tumor types, being upregulated in HGG but reduced in MPNST, particularly NF1-associated cases, suggesting that its biological role and potential as a therapeutic target may be context-dependent. We found that LGG as a whole had lower median RECQL4 H-scores than HGG. Taken alone, both LGDC or LGCG showed a significantly lower expression if compared to HGG. However, by stratifying for different types of LGCG analyzed, no significant differences are noted in terms of RECQL4 protein expression.

ALT-negative HGGs typically rely on telomerase for tumor survival and immortalization. In our cohort, ALT-positive and negative HGGs did not have statistically significant differences regarding RECQL4 expression. Of note, a possible relationship between RECQL4 upregulation and *TERT* alterations has been extensively investigated in preclinical settings.^6^ Namely, HRQ1, the yeast equivalent of human RECQL4, stimulates telomerase activity in *S. cerevisiae*,^15^ and inversely, TERT was observed to rely on RECQL4 for its immortalization activity to be effective in an in vitro model of cultured fibroblasts from a family with germline *RECQL4* variants.^16^ However, the hypothesis that RECQL4 overexpression is a direct consequence of TERT alterations is currently unproven and is not reflected in our data pertaining HGGs. A potential clinical value of RECQL4 lies in its possible prognostic and predictive significance in human cancer, particularly gliomas and MPNST. In this view, to pinpoint the precise biological type of alteration in *RECQL4* that harbors such critical value (protein upregulation versus downregulation) is critical. To date, published data demonstrate variable results. Regarding survival in the clinical setting, a study found a correlation between two polymorphisms in introns of *RECQL4* in glioblastoma, but no association of a non-synonymous SNP in the same gene.^17^ Another study reported an association between high levels of RECQL4 (mRNA and protein) and worse survival.^10^ Regarding the predictive value of RECQL4, its depletion in glioma cells conferred increased response to cytotoxic agents.^10^

The possible clinical value of RECQL4 loss of function was explored in our cell line analyses. We found slightly decreased survival in RECQL4-knockdown glioma/MPNST cell lines due to increased apoptosis and a significantly increased susceptibility of such cell lines to ATR-kinase inhibitors. In U251 and NF90-8 cell lines, AZD6738 effect was enhanced in *RECQL4-*knockdown cells in comparison to pLKO.1 controls. Such difference was not evident in ST88-14 cell lines. Notably, the latter lacks mutations in *TP53*, which may raise the possibility of a role for such alterations in AZD6738 susceptibility, particularly in the setting of *NF1* mutations.

Taken together, our data suggest that RECQL4 plays a role in the biology of gliomas and MPNSTs, with higher expression in high-grade gliomas and lower expression in MPNSTs, particularly NF1-associated cases. While *RECQL4* mutations are rare, a subset of those mutations are potentially of loss-of-function significance. However, increased protein expression correlates with tumor grade in gliomas and may reflect distinct mechanisms of telomere maintenance. Functionally, *RECQL4* knockdown sensitizes tumor cells to ATR inhibition, suggesting a potential therapeutic vulnerability in the clinical setting and possibly, a diagnostic role of RECQL4 IHC for treatment selection. These findings support further investigation of RECQL4 as both a biomarker and target in select tumor contexts.

This study has several limitations. The analysis of the prevalence of *RECQL4* variants was performed for coding alterations and not for other types of variants (eg deep deletions) and did not consider critical factors such as IDH1/2 status, *MGMT* promoter methylation, and other molecular and clinical aspects for cases that underwent NGS. The median scores of different types of glioma, neurofibroma, and MPNST TMAs showed a great degree of overlap; however, the results are in line with previous reports. More detailed functional and bioinformatic studies can be also helpful to clarify any relationship between *RECQL4* alterations and specific molecular signatures (eg HRD) reflecting genomic instability.

More research is needed to clarify: (1) the mechanisms of RECQL4 upregulation; (2) the relationship of such mechanisms to chemo/radiotherapeutic and targeted therapy agents; and (3) the role of RECQL4 IHC in the clinical setting.

## Supplementary Material

nlaf129_Supplementary_Data
