# Supplementary material for: RECQL4 alterations in gliomas and nerve sheath tumors: Expression patterns and therapeutic implications
Source: J Neuropathol Exp Neurol. 2025 Nov 29;84(12):1191–201. doi: 10.1093/jnen/nlaf129 (PMC12713544; doi:10.1093/jnen/nlaf129)

Supplementary Figure S1. Oncoprint representation of *RECQL4* alterations in gliomas in cBioPortal. Visualization of *RECQL4* genetic alterations across 10 different studies included in cBioPortal (4206 samples, 3453 patients); alterations include missense mutations, truncating mutations, splice variants, and copy number changes.

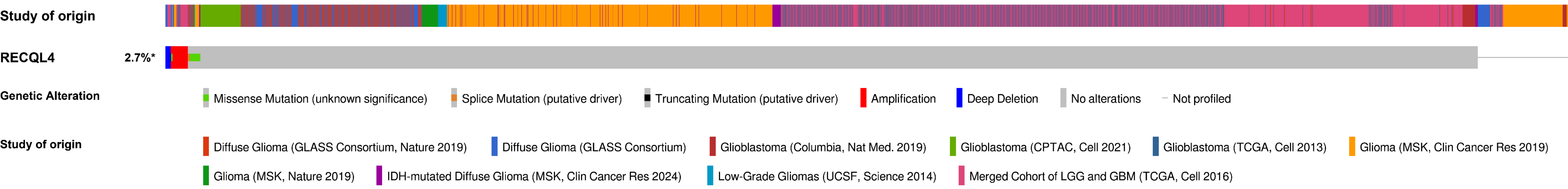

Supplementary Figure S2. Breakdown of the 10 glioma studies in cBioPortal analyzed for *RECQL4* alterations, including number of cases per study and study identifiers. Alterations are color coded and include coding mutations, amplifications, and deep deletions.

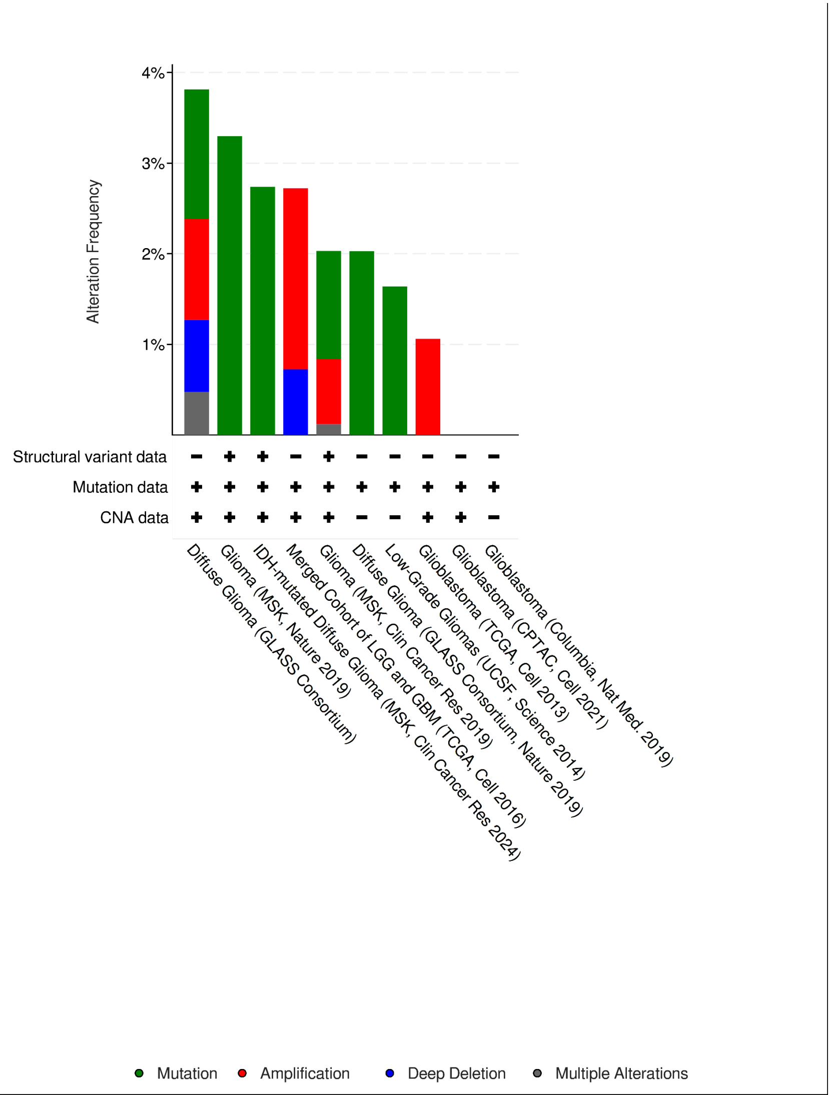

Supplementary Figure S3. Distribution of *RECQL4* alterations by glioma subtype in cBioPortal. Frequency and type of *RECQL4* genetic alterations stratified by glioma subtype. Alterations are color coded and include coding mutations, amplifications, and deep deletions.

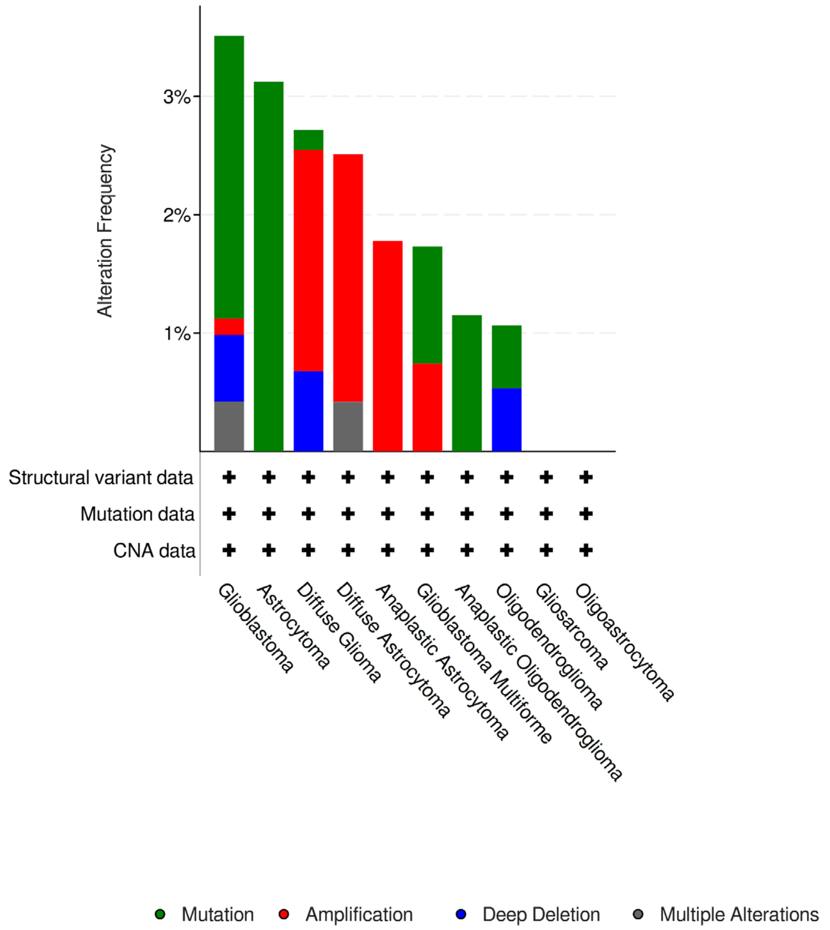

Supplementary Figure S4. Lollipop plot showing different types of *RECQL4* coding mutations identified in gliomas in cBioPortal. Detailed annotation of prevalence and different types of 59 coding variants found in gliomas on cBioPortal data, including 5 predicted pathogenic variants (1 truncating, 4 splice site) and 54 variants of unknown significance (missense). Alterations are color coded.

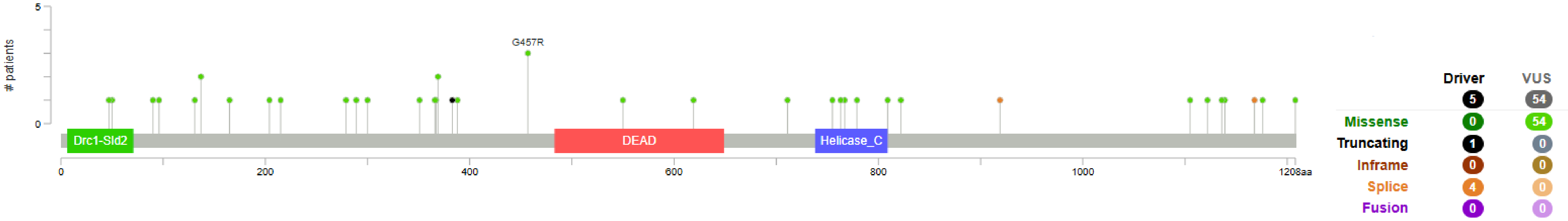

Supplementary Figure S5. Overall survival analysis by *RECQL4* alteration status in glioma in cBioPortal. Kaplan–Meier survival curves comparing glioma patients with RECQL4-altered tumors (red line) to those with *RECQL4*-wildtype tumors (blue line). *RECQL4* alterations are associated with longer overall survival (median OS: 65.0 vs. 25.5 months; Log-rank P = 0.006).

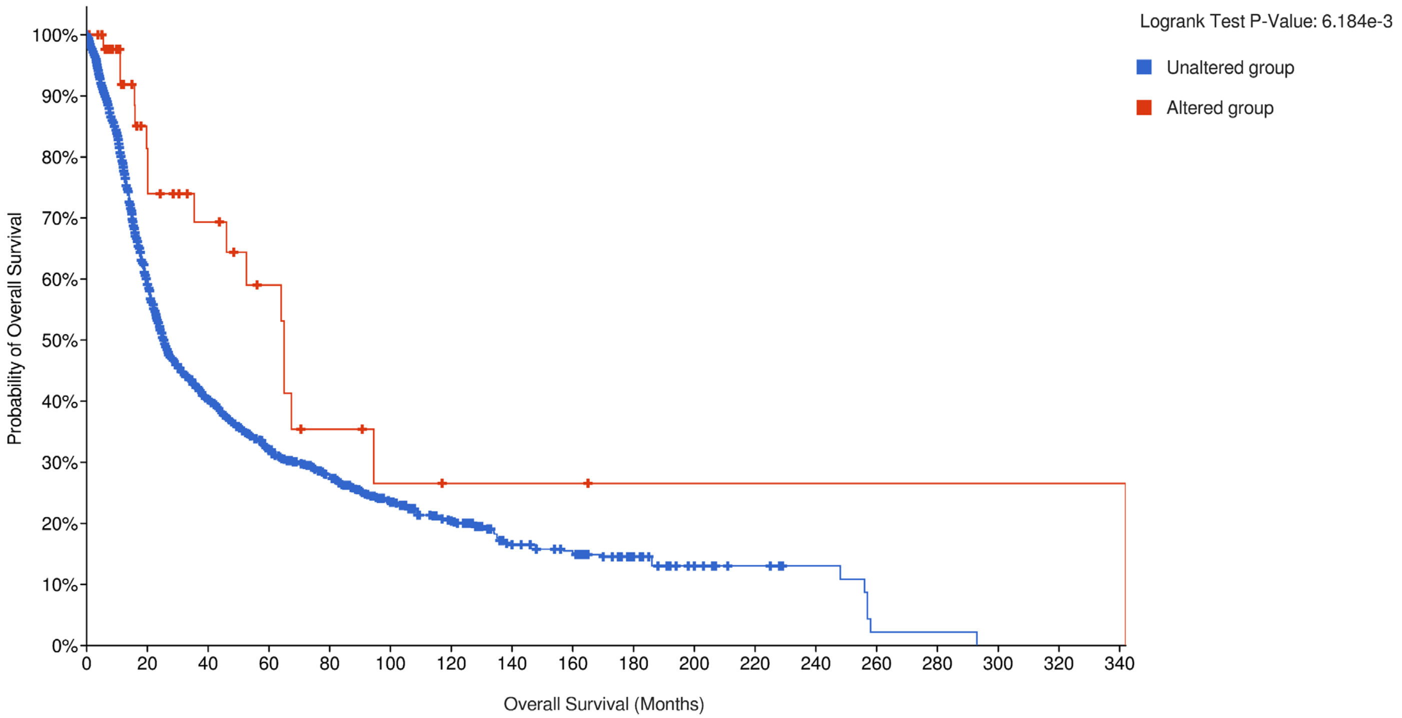

Number at risk (n)

Altered group	48	22	15	10	5	3	2	2	2	1	1	1	1	1	1	1	1	1
Unaltered group	2867	1168	640	412	290	186	122	73	53	33	17	10	6	1	1	0	0	0

Supplementary Figure S6. RECQL4 immunohistochemical H-scores in adult-type high-grade glioma, stratified by IDH status, ALT status, and ATRX status. No statistical significance was observed in all instances, calculated using the Wilcoxon rank-sum test (two-sided).

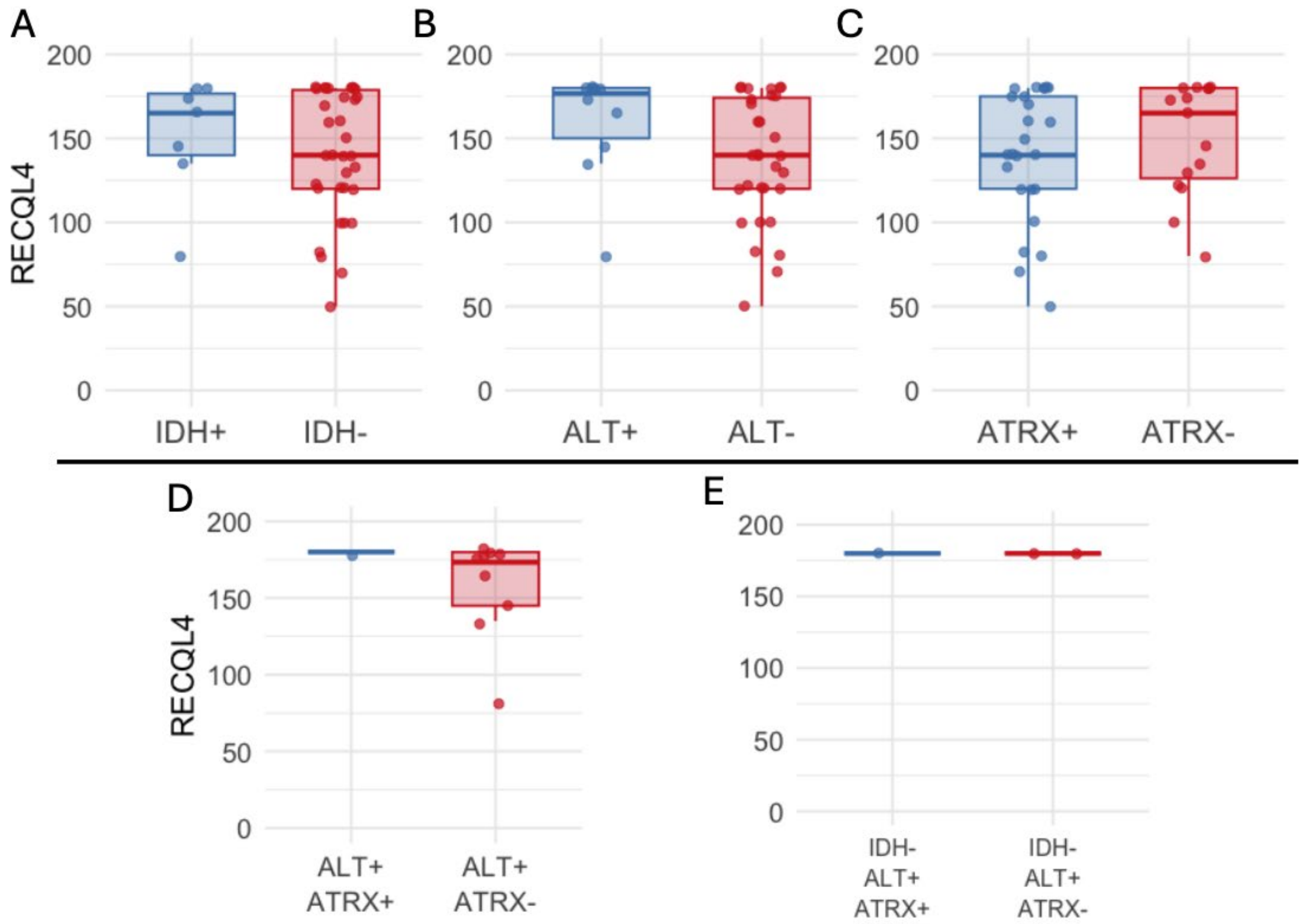

Supplementary figure S7. Graphical abstract of RECQL4 alterations in CNS tumors study.

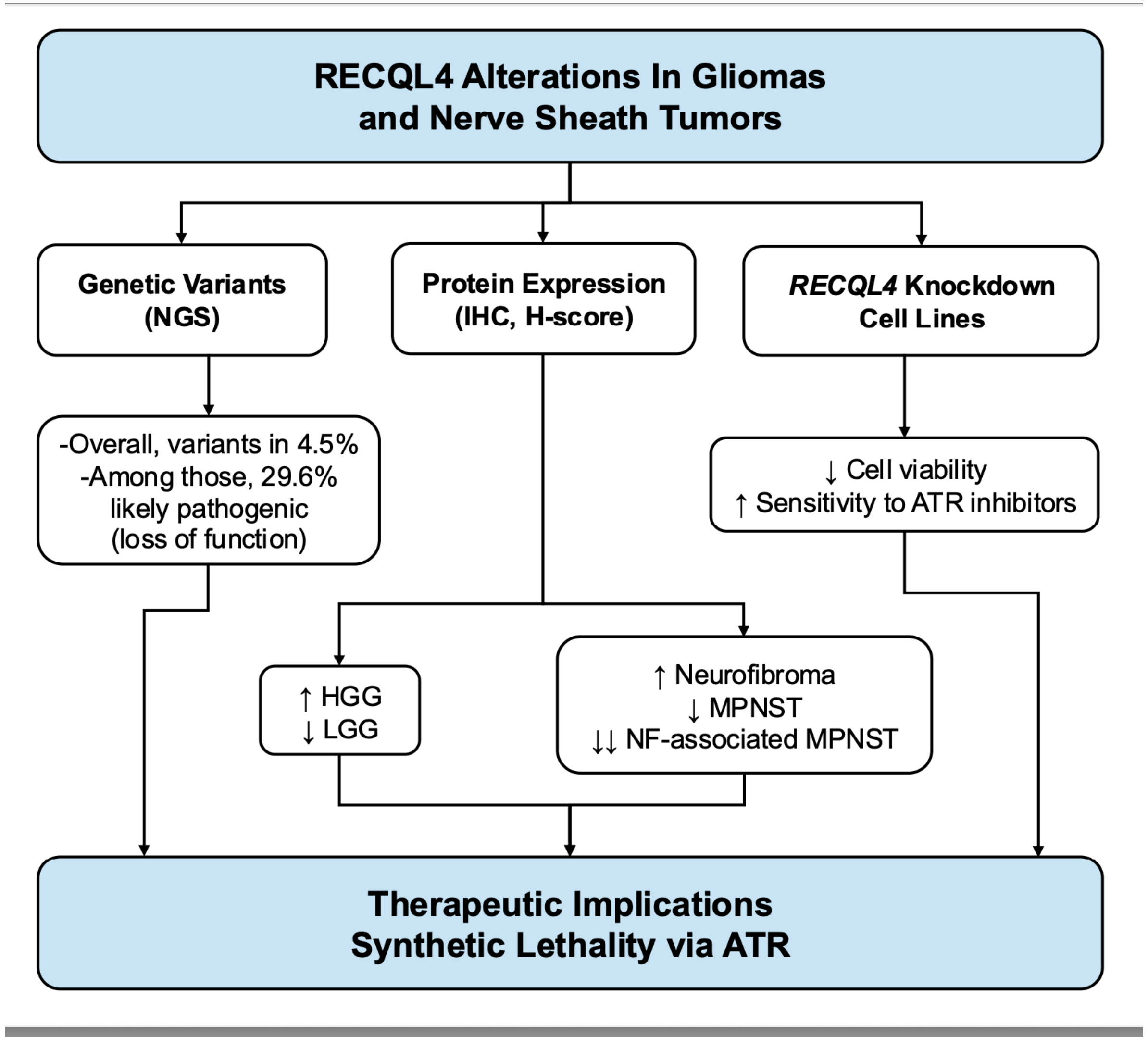

Supplement: nlaf129_Supplementary_Data [file nlaf129_supplementary_data.zip › SUPPLEMENTARY FIGURES WITH LEGENDS FILE.pdf]
